# Enhancer-driven chromatin interactions during development promote escape from silencing by a long non-coding RNA

**DOI:** 10.1186/1756-8935-4-21

**Published:** 2011-11-15

**Authors:** Lisa Korostowski, Anjali Raval, Gillian Breuer, Nora Engel

**Affiliations:** 1 Fels Institute for Cancer Research & Molecular Biology & Department of Biochemistry, Pharmacy Building, Room 201, Temple University School of Medicine, Philadelphia, PA 19104, USA

**Keywords:** Imprinting, non-coding RNAs, *Kcnq1ot1*, *Kcnq1*, chromosome conformation capture (3C)

## Abstract

**Background:**

Gene regulation in eukaryotes is a complex process entailing the establishment of transcriptionally silent chromatin domains interspersed with regions of active transcription. Imprinted domains consist of clusters of genes, some of which exhibit parent-of-origin dependent monoallelic expression, while others are biallelic. The *Kcnq1 *imprinted domain illustrates the complexities of long-range regulation that coexists with local exceptions. A paternally expressed repressive non-coding RNA, *Kcnq1ot1*, regulates a domain of up to 750 kb, encompassing 14 genes. We study how the *Kcnq1 *gene, initially silenced by *Kcnq1ot1*, undergoes tissue-specific escape from imprinting during development. Specifically, we uncover the role of chromosome conformation during these events.

**Results:**

We show that *Kcnq1 *transitions from monoallelic to biallelic expression during mid gestation in the developing heart. This transition is not associated with the loss of methylation on the *Kcnq1 *promoter. However, by exploiting chromosome conformation capture (3C) technology, we find tissue-specific and stage-specific chromatin loops between the *Kcnq1 *promoter and newly identified DNA regulatory elements. These regulatory elements showed *in vitro *activity in a luciferase assay and *in vivo *activity in transgenic embryos.

**Conclusions:**

By exploring the spatial organization of the *Kcnq1 *locus, our results reveal a novel mechanism by which local activation of genes can override the regional silencing effects of non-coding RNAs.

## Background

Genomic imprinting is a transcriptional regulatory mechanism that results in parental-specific gene expression. Over the past two decades, many mechanistic insights have emerged from the study of such loci as the *H19*/*Igf2 *domain [1]. However, most imprinted loci are much more complex and exhibit tissue-specific as well as stage-specific imprinting. Many significant questions remain concerning the regulatory mechanisms governing such extended domains. For example, how genes that are monoallelic can coexist interspersed with others that exhibit partial or full biallelic expression is still not understood. The prevalent hypotheses for the appearance of imprinting imply that, although advantageous in some respects, it imposed a burden on bystander genes that came under its influence. Thus, it is likely that mechanisms emerged to bypass the effects of allelic silencing.

**Figure 1:**
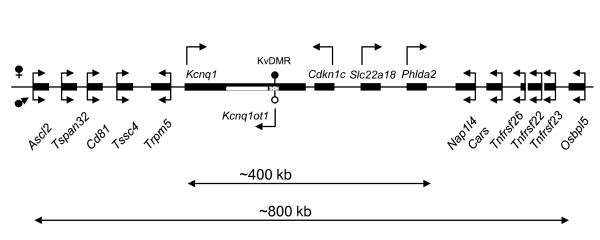
**Schematic of the *Kcnq1 *imprinted domain on mouse chromosome 7**. The imprinting pattern in the embryo is shown. Arrows indicate direction of transcription. Arrows above the genes represent maternal transcription, arrows below the line are paternal transcription and genes with two arrows have biallelic expression.

The *Kcnq1 *domain consists of at least ten genes exhibiting parental allele-specific expression [2], interspersed with five genes that are biallelically expressed. The key regulatory element, KvDMR, is a CG-rich promoter for a long, non-coding RNA [3]. The paternal copy of the KvDMR is hypomethylated and active, resulting in production of a 90-kb non-coding RNA (ncRNA), *Kcnq1ot1*. Transcription of the *Kcnq1ot1 *RNA has *cis*-silencing effects on the neighboring genes, spanning a region of 750 kb in the placenta and 400 kb in the embryo. The KvDMR is methylated on the maternal allele and as a consequence, maternal promoter activity is inhibited. As the ncRNA is not produced maternally, most genes within the domain are free to be expressed from that chromosome (Figure 1).

Recent experiments have strongly suggested that the silencing mechanism of *Kcnq1ot1 *RNA involves a spreading activity in *cis*, recruitment of Polycomb group proteins and physical compaction of the whole domain [4-6]. However, the presence of the ncRNA is not uniform throughout the region [4], leaving open the question of how this relates to biallelic expression of some genes.

Genes such as *Trpm5 *are constitutively biallelic both in the embryo and placenta. The *Kcnq1 *gene has a more complex pattern: it is monoallelic and ubiquitously expressed in early embryos, but the paternal copy is activated in conjunction with the acquisition of a tissue-restricted expression pattern established by mid gestation, that is, in the heart, kidney and brain [7]. Thus, the *Kcnq1 *domain illustrates the regulatory challenges that must be met in a complex imprinted domain.

How do genes such as *Kcnq1 *achieve tissue-specific escape from imprinting? One possible explanation is that the *Kcnq1 *promoter is exceptionally strong and that as tissue-specific factors become expressed and bind to it, the silencing effect of *Kcnq1ot1 *is overcome. Alternatively, tissue-specific enhancers that become active may override the effects of ncRNAs. An additional possibility is that boundary elements serving as barriers to the spread of the ncRNA may exist within the *Kcnq1 *gene. These proposed mechanisms are not mutually exclusive.

None of the regulatory elements that account for the complex patterns of gene expression in this region have been identified. We used an optimized approach for identifying novel regulatory DNA elements and to determine their role in promoting escape from silencing. We investigated the mechanism of *Kcnq1 *reactivation by determining the *in vivo *spatial organization of the *Kcnq1 *domain with chromosome conformation capture (3C) assays [8,9]. We present evidence for the role of specific DNA interactions by chromatin looping as a mechanism for acquisition of tissue-specific expression. These contacts occur during the developmental window in which the paternal allele of *Kcnq1 *escapes silencing by *Kcnq1ot1*. To determine if the regions contacted predominantly by the *Kcnq1 *promoter are regulatory elements, we integrated comparative genomics data and publicly available genome-wide chromatin immunoprecipitation (ChIP) profiles and identified DNA sequences that are candidates for modulating gene activity. Candidates were tested *in vitro *and *in vivo *and several elements were identified as active transcriptional enhancers. Their role in overriding RNA-mediated repression is discussed.

## Results

### *Kcnq1 *RNA transitions from monoallelic to biallelic expression during development

**Figure 2:**
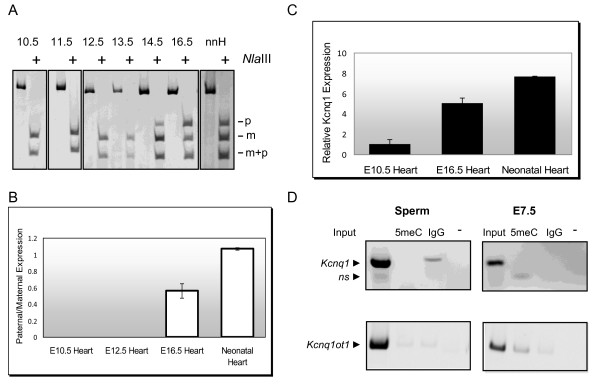
**(A) Developmental imprinting pattern of *Kcnq1***. Allele-specific expression of *Kcnq1 *as assayed by reverse transcription (RT)-PCR and restriction digest with *Nla*III on E10.5, 11.5, 12.5, 13.5, 14.5, 16.5 and neonatal heart (nnH) from F1 hybrid B6(CAST7) × C57BL/6J crosses. Digestion products specific for B6(CAST7) (maternal) and C57BL/6J (paternal) alleles are indicated. Positive signs (+) denote addition of *NlaIII *to the RT-PCR product. **(B) **Quantification of relative paternal-specific and maternal-specific expression during development. **(C) ***Kcnq1 *RNA abundance during stages of development in which the imprinting pattern switches from monoallelic to biallelic, as assayed by real-time PCR. **(D) **Methylated DNA immunoprecipitation (MeDIP) analysis of the *Kcnq1 *and *Kcnq1ot1 *promoter regions in sperm and 7.5 days post coitum (dpc) embryos. 5meC lane = DNA precipitated by antibody against methylated cytosine; IgG = non-specific immunoprecipitation; Input = DNA before immunoprecipitation; - = no antibody control. Specific bands for *Kcnq1 *and *Kcnq1ot1 *are indicated; NS = non-specific amplification product. The *Kcnq1ot1 *promoter is methylated maternally in 7.5 dpc embryos and unmethylated in sperm, thus serving as a positive control for immunoprecipitation of methylated DNA in E7.5 DNA and a negative control in sperm DNA.

Congenital long QT syndrome type I is a cardiac disorder in which defects in KCNQ1, a voltage-gated potassium channel, result in serious cardiac arrhythmias [10]. There is a wide range of phenotypes, with some individuals remaining mostly asymptomatic and others presenting severe symptoms. Understanding the epigenetic profile of *Kcnq1 *expression during cardiac development will aid in understanding the molecular mechanisms underlying the phenotypic variability. We determined *Kcnq1 *expression levels during development to pinpoint the exact timing of the switch from monoallelic to biallelic expression (Figure 2A). Allele-specific reverse transcription (RT)-PCR showed that *Kcnq1 *switched from a monoallelic to biallelic pattern between E13.5 and E14.5. Expression emanating from the paternal allele became progressively stronger, reaching levels equal to the maternal allele (Figure 2B). To determine whether the activation of the paternal *Kcnq1 *allele was accompanied by an increase in total *Kcnq1 *RNA abundance, quantitative (q)PCR on total RNAs from these stages was performed. Whereas the transition from monoallelic to biallelic expression is expected to double RNA abundance, the total cellular RNA levels increased eightfold (Figure 2C), suggesting that other enhancing mechanisms are involved in achieving the final RNA levels.

One possible explanation for escape of silencing by the paternal *Kcnq1 *allele is that expression is initiated from an alternative start site. However, we did not observe any alternative transcripts after the transition from monoallelic to biallelic expression, thus excluding this hypothesis (data not shown). We then speculated that, because the transcriptional start site (TSS) at exon 1 lies within a strong CpG island, it could be paternally methylated in the early embryo and become demethylated upon activation. To determine if the CpG island was methylated during spermatogenesis or early embryogenesis, we performed methylated DNA immunoprecipitation (MeDIP) on sperm and embryos at 7.5 days post coitum (dpc) (Figure 2D). No DNA methylation was detected on the paternal allele, suggesting that the promoter is protected from primary (gametic) and secondary (post fertilization) methylation, even though it is silent at these stages. Furthermore, as opposed to the secondary methylation of the upstream *Cdkn1c *gene orchestrated by *Kcnq1ot1*, the silencing effect of the ncRNA on *Kcnq1 *is not mediated by DNA methylation.

### *Kcnq1 *exhibits tissue-specific changes in chromatin conformation

**Figure 3:**
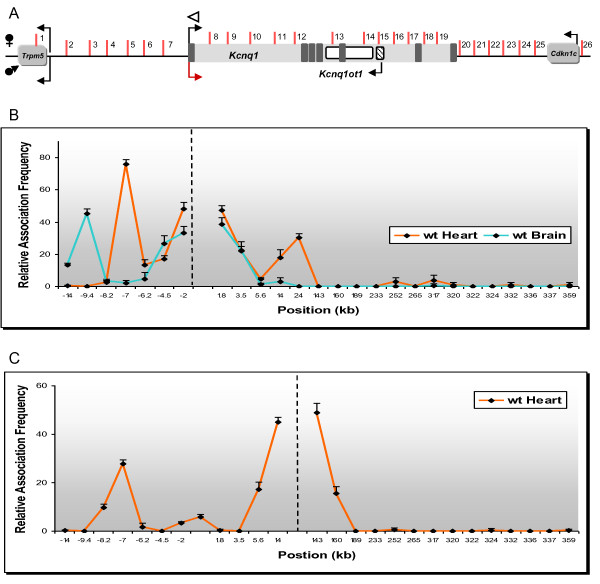
**Chromosome conformation capture (3C) mapping of long-range chromatin interactions in the *Kcnq1 *region in neonatal tissues**. **(A) **Genomic organization of the *Kcnq1 *region scanned in this study, spanning 387 kb. Dark boxes in the *Kcnq1 *gene are exons. Arrows show direction of transcription; maternal-specific and paternal-specific expression is indicated above and below the line. Reactivated *Kcnq1 *expression is depicted as a red arrow. Vertical bars indicate only the *Afl*III/*Nco*I restriction sites analyzed in this study, with the fragments assayed by the variable primers for contact with the *Kcnq1 *promoter numbered. White arrowhead indicates the anchor primer at the *Kcnq1 *promoter. Graphical representation is not to scale. **(B,C) **Relative crosslinking frequency of regions interacting with the *Kcnq1 *promoter, with association values plotted on the y-axis. Distances in kb relative to the *Kcnq1 *promoter are plotted along the x-axis (not to scale). Each value is derived from three independent samples and the standard error is indicated. Hatched vertical line represents the anchor position. High crosslinking frequencies with the anchor fragment indicate close proximity. (B) 3C map of neonatal heart and brain. (C) Reciprocal 3C analysis of heart-specific interactions in the *Kcnq1 *region by anchoring the PCR reactions at fragment 12 (intron 1).

Three-dimensional conformation of chromatin plays a crucial role in bringing widespread regulatory elements into close proximity [11-13]. 3C technology detects interactions between DNA regions by *in vivo *crosslinking of chromatin and PCR with primers specific for novel junctions created by ligation of sequences that were in close proximity through specific interactions. To determine if the *Kcnq1 *promoter associated with specific DNA sequences in the neonatal heart, we performed an unbiased, systematic 3C scan extending 12.6 kb upstream of the promoter, spanning the 320 kb *Kcnq1 *gene and 31.3 kb downstream of the last exon. Our strategy was to anchor the 3C assays with an invariant primer at the *Kcnq1 *promoter (Figure 3A, white arrowhead) and variable primers located in restriction fragments across the domain (Figure 3A, fragments tested are numbered from 1 to 26). Strong and highly specific interactions were detected between the *Kcnq1 *promoter and fragment 4 (Figure 3B, orange line, peak at -8 kb upstream of *Kcnq1*). Additionally, there were weaker but specific contact with fragments 11 and 12 (Figure 3B, orange line, peak in intron 1).

To see if this profile was tissue specific, we performed 3C in neonatal brain, a tissue in which *Kcnq1 *is highly expressed and also biallelic. The interaction profile observed in the brain was distinct from that of the heart. The *Kcnq1 *promoter strongly associated instead with fragment 2 (Figure 3B, blue line, peak at -12 kb) and showed no contact with fragment 12 in the intron. These data show that there are distinct three-dimensional conformations that regulate expression in each tissue and identify the contact regions as putative tissue-specific enhancers.

**Figure 4:**
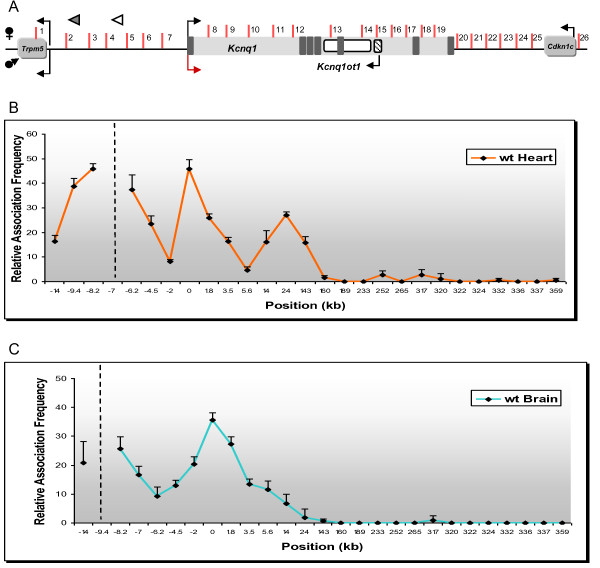
**Reciprocal chromosome conformation capture (3C) scans anchoring at evolutionarily conserved regions**. **(A) **Genomic organization of the *Kcnq1 *region scanned in this study, spanning 387 kb. Dark boxes in the *Kcnq1 *gene are exons. Arrows show direction of transcription; maternal-specific and paternal-specific expression is indicated above and below the line. Reactivated *Kcnq1 *expression is depicted as a red arrow. Vertical bars indicate the *Afl*III/*Nco*I restriction sites analyzed in this study, with the fragments assayed with the variable primers for contact with the tissue-specific evolutionarily conserved regions (ECRs) numbered. White arrowhead, anchor primer for neonatal heart; gray arrowhead, anchor primer for neonatal brain. Graphical representation is not to scale. **(B) **Relative crosslinking frequency of regions interacting with ECR 4. The peaks correspond to the *Kcnq1 *promoter and fragment 12 within intron 1. **(C) **Relative crosslinking frequency of regions interacting with ECR 2. The peaks correspond to the *Kcnq1 *promoter and fragment 12 within intron 1. Hatched vertical lines represent the anchor position.

These results were verified by carrying out the reciprocal analysis, that is, 3C assays were anchored with a primer in fragment 12 of intron 1 (Figure 3C, white arrowhead at +24 kb). In the neonatal heart, we found increased association between this region and the *Kcnq1 *promoter, but also with fragment 4, at -8 kb upstream. We did not observe these contact points in neonatal brain (data not shown). In addition, reciprocal analyses with anchors at fragment 4 in neonatal heart (Figure 4B) and at fragment 2 in neonatal brain (Figure 4C) confirmed the interactions between these sequences with the *Kcnq1 *promoter region, underscoring the presence of tissue-specific conformations.

### Onset of loop formation between the *Kcnq1 *promoter and putative enhancers correlates with the monoallelic to biallelic switch

**Figure 5:**
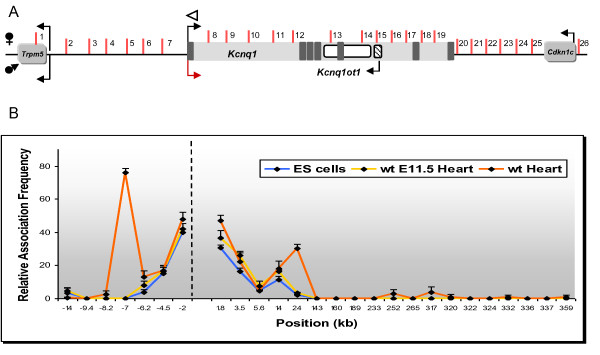
**Developmental profile of chromatin interactions in the *Kcnq1 *region**. **(A) **Genomic organization of the *Kcnq1 *region scanned in this study, spanning 387 kb. Dark boxes in the *Kcnq1 *gene are exons. Arrows show direction of transcription; maternal-specific and paternal-specific expression is indicated above and below the line. Reactivated *Kcnq1 *expression is depicted as a red arrow. Vertical bars indicate only the *Afl*III/*Nco*I restriction sites analyzed in this study, with the fragments assayed with the variable primers for contact with the *Kcnq1 *promoter numbered. White arrowhead indicates the anchor primer at the *Kcnq1 *promoter. Graphical representation is not to scale. **(B) **Developmental profile of interactions of the *Kcnq1 *promoter in ES cells, 11.5 days post coitum (dpc) heart, and neonatal heart. Relative crosslinking frequency of regions interacting with the *Kcnq1 *promoter, with association values plotted on the y-axis. Distances in kb relative to the *Kcnq1 *promoter are plotted along the x-axis (not to scale). Each value is derived from three independent samples and the standard error is indicated. Hatched vertical line represents the anchor position. High crosslinking frequencies with the anchor fragment indicate close proximity.

We next looked for dynamic changes in the status of the chromatin fiber related to the escape from silencing of *Kcnq1 *by testing the conformation in E11.5 hearts. At this stage, the paternal *Kcnq1 *allele is still silent (Figure 5). None of the interactions evident in the neonatal heart and brain between the *Kcnq1 *promoter and upstream regions was apparent in the embryos. However, there was a significant physical association of the promoter with a region in intron 1, though restricted to fragment 11 (Figure 5B, yellow line). Thus, monoallelic expression is not dependent on contacts between the promoter and the putative upstream enhancers. However, onset of loop formation with those regions does correlate with the monoallelic to biallelic switch.

### Distinct developmental history of potential regulatory elements at the *Kcnq1 *locus

To expand the epigenetic profile of *Kcnq1 *to earlier developmental stages, we performed locus-wide 3C assays on mouse ES cells (Figure 5B, blue line). None of the heart-specific contacts between the *Kcnq1 *promoter and regions upstream were detected, but the interaction with fragment 11 in intron 1 was already present. Thus, 3C assays show an interaction between the *Kcnq1 *promoter and a region in intron 1 that is already apparent in early stages of embryogenesis and becomes progressively stronger as development ensues. Additionally, the *Kcnq1 *promoter associates with upstream elements concomitantly with the transition from monoallelic to biallelic expression. These upstream elements partner with the *Kcnq1 *promoter in a tissue-specific fashion.

### Sequences identified by contact analysis have enhancer activity *in vitro *and *in vivo*

**Figure 6:**
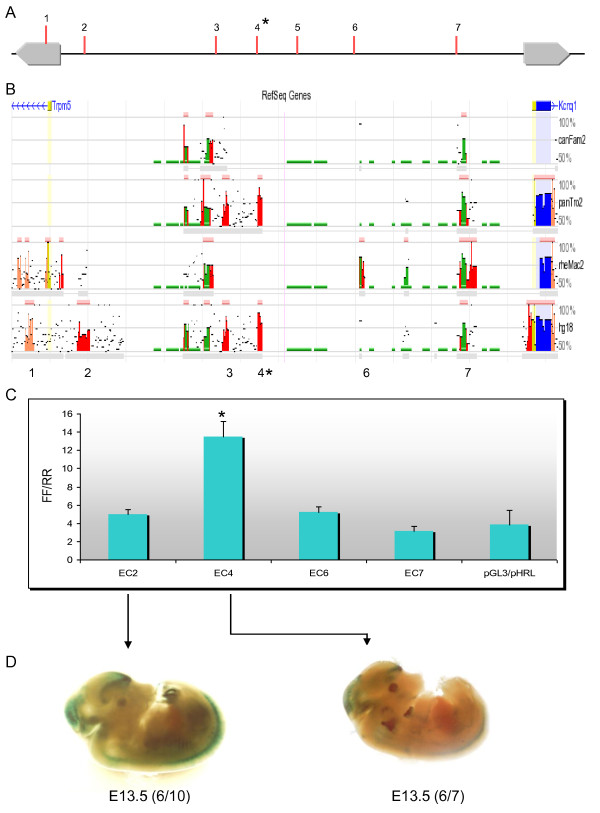
**Identification and functional validation of candidate regulatory elements**. **(A) **Representation of the upstream region of *Kcnq1*, indicating the fragments tested in the chromosome conformation capture (3C) assays. **(B) **Graphic display of the conservation profiles for the upstream region of *Kcnq1*. The base genome is mouse. Evolutionarily conserved regions (ECRs) of a minimum of 100 bp conserved above 70% sequence identity are displayed as red (intergenic) peaks, with the x-axis representing positions in the base genome and the y-axis representing percentage identity between the base and the aligned genomes. Annotated genes are depicted in blue. Numbered peaks represent the fragments tested for enhancer activity. **(C) **DNA sequences were inserted in pGL3-promoter vector upstream of the luciferase reporter. Luciferase activity was normalized to *Renilla *activity. All transfections were performed in triplicate. The asterisk denotes the interaction observed in neonatal heart by 3C. **(D) **Representative LacZ-stained embryos with *in vivo *enhancer activity. ECR4 and ECR2 exhibit tissue-specific activity, with numbers showing the reproducibility of LacZ reporter staining.

A search of publicly available databases including the VISTA enhancer browser [14] revealed an intragenic element (mm183) with a heart-specific expression pattern in 11.5 dpc transgenic mouse embryos. This element lies in fragment 10, immediately upstream of the sequence contacted by the promoter in intron 1. This strongly supports its role in the development of heart-specific transcription of *Kcnq1*. Examination of conservation profiles for the *Kcnq1 *region from the dCODE website (http://www.dcode.org) [15] revealed discrete evolutionarily conserved regions (ECRs) (parameters set to 70% identity with a minimum length of 100 bp) that were contained on restriction fragments that had been tested in the 3C substrates (Figure 3). By combining *in silico *analysis and published epigenomic profiles, including chromatin status [16] and occupancy of p300 [17], we selected ECRs that were good candidates for enhancers, focusing on the intergenic region upstream of the *Kcnq1 *gene, numbering them according to the 3C fragments they overlapped (Figure 6A,B). None of these ECRs were conserved in opossum, chicken, frog or fish.

To determine whether these sequences had enhancer activity *in vitro*, we cloned the DNA fragments upstream of a luciferase reporter gene and tested enhancer activity by transient transfection into NIH-3T3 cells (Figure 6C). ECR 4, contained in fragment 4 that showed frequent colocalization with the *Kcnq1 *promoter in neonatal heart, had the most pronounced enhancer effect *in vitro*. ECR 2, which associated with the *Kcnq1 *promoter in the neonatal brain, did not show significant transcriptional activity in this assay, suggesting that fibroblasts may not contain the factors required for brain-specific activity.

We next cloned several candidate sequences into an enhancer reporter vector and transgenic mouse embryos were generated. Several independent embryos were assessed for LacZ activity at 9.5 and 13.5 dpc. Reproducible staining in hindbrain, forebrain and thoracic region was observed with ECR4 at 13.5 dpc (Figure 6D), but none at 9.5 dpc (data not shown), recapitulating the developmental expression pattern of *Kcnq1*. ECR2 directed expression to hindbrain and forebrain in the 13.5 dpc embryos.

## Discussion

Our data reveal a novel role for transcriptional enhancers, namely overcoming regional imprinting effects to permit escape from silencing and leading to biallelic expression. Thus, the evolution of enhancers in the mammalian genome to enable biallelic expression of some genes located near imprinted genes appears to be an essential adaptation to accommodate imprinting as a regulatory mechanism. By mapping the architecture adopted by a specific genomic region during development, we have found dynamic changes that counter the regional repressive effect of a long non-coding RNA, *Kcnq1ot1*. Specifically, escape from silencing of the *Kcnq1 *gene is accomplished by the activation of tissue-specific enhancers and involves local reorganization of higher order structure.

The biallelic expression of genes within the *Kcnq1 *domain is reminiscent of certain genes on the × chromosome that escape inactivation [18,19], but differs in significant ways. *Kcnq1 *is initially silenced paternally but transitions to biallelic expression at a specific time point during development. In theory, tissue-specific enhancer activity could override silencing by *Kcnq1ot1 *by physically contacting the promoter and sequestering it from condensing factors recruited by the ncRNA. In fact, the data from the 3C assays that we present are highly supportive of such a model by showing that stage and tissue-specific conformations of chromatin bring the *Kcnq1 *promoter into physical contact with DNA sequences that are highly conserved and exhibit enhancer activity *in vivo*.

In fact, we could distinguish two types of contacts. Promoter interaction with an element in intron 1 (mm183) is already apparent in early stages of embryogenesis and becomes progressively stronger and broader as development ensues. This association foreshadows a later event in which the *Kcnq1 *promoter contacts tissue-specific upstream elements concomitantly with the reactivation of the paternal allele and the restriction of expression to specific organs. Indeed, previous studies on the chromatin marks in the region showed that, in contrast to other neighboring genes, the *Kcnq1 *promoter does not exhibit differential histone modifications associated with repression [20,21], and our data indicate that it is also devoid of DNA methylation on the paternal allele. We speculate that the intragenic enhancer may be marked in early embryogenesis by pioneer factors that maintain an open, poised chromatin status [22] and prevent assembly of repressive factors associated with the spreading of *Kcnq1ot1 *[4,5].

**Figure 7:**
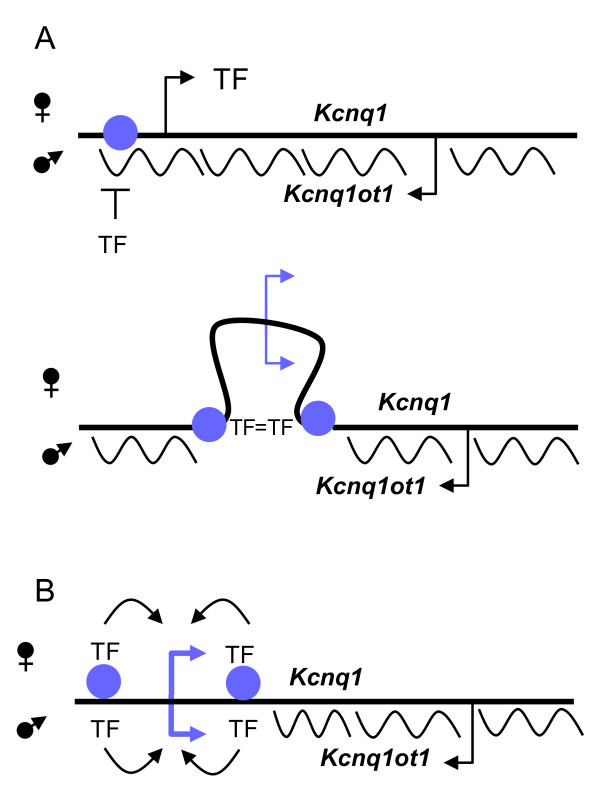
**Model for escape from silencing by the *Kcnq1 *gene**. Enhancer candidates, depicted as purple circles, become active upon binding tissue-specific transcription factors (TF); ncRNA, wavy lines. **(A) **Appearance of enhancer-specific transcription factors promotes a conformational change that sequesters the *Kcnq1 *promoter from the effects of *Kcnq1ot1*. **(B) **Factors binding in intron 1 of *Kcnq1 *act as a boundary for the *Kcnq1ot1 *ncRNA on the paternal allele, leaving it available for tissue-specific activation by enhancer-binding regulatory proteins.

We propose that as development proceeds, both the upstream and downstream contacts evident in the 3C assays promote a conformation that excludes the *Kcnq1 *promoter from the influence of the *Kcnq1ot1 *ncRNA by establishing an autonomous regulatory loop (Figure 7A). An alternative is that tissue-specific enhancer-driven expression can override the silencing biochemically, by countering the chromatin condensation directed by *Kcnq1ot1 *(Figure 7B). In both cases, the DNA elements involved are altering chromatin conditions locally, thereby limiting a global long-range silencing mechanism; in other words, they are establishing a boundary. It will be crucial now to determine the DNA-binding factors that mediate this activity and the effect that deleting these regions has on the expression and imprinting patterns of *Kcnq1 *and neighboring genes. It will also be important to test if mutations that eliminate *Kcnq1ot1 *expression can affect the remodeling of the *Kcnq1 *locus during development. Additionally, it will be interesting to see whether genes silenced by different epigenetic mechanisms, either during development or in disease states, are governed by similar conformational determinants.

## Conclusions

In summary, we have explored the organization of the *Kcnq1 *region in three-dimensional nuclear space during development and have identified DNA sequence elements that achieve the dual purpose of reactivating a gene silenced by a non-coding RNA and directing tissue-specific expression. Our studies underscore the importance of providing a three-dimensional context to the information on DNA methylation and chromatin modifications in order to fully grasp the dynamics of gene regulation in different cell lineages during development.

## Methods

### Ethics

This study was carried out on mice in strict accordance with the recommendations in the Guide for the Care and Use of Laboratory Animals of the National Institutes of Health. The protocol was approved by the Temple University Animal Care and Use Committee (Protocol 3294).

### RNA purification

Heart tissues for total RNA extraction were collected at appropriate days of gestation using F1 hybrid mice from C57BL/6J × B6(CAST7) crosses from embryonic to neonatal stages. RNA was extracted using TRIzol Reagent (Invitrogen, Carlsbad, CA no. 15596-018) and following manufacturer's protocol for RNA extraction from tissues. All RNA samples were subjected to DNase treatment using Turbo DNA-free (Ambion, Austin, TX no. AM1907) using the rigorous DNase treatment protocol. Three to five biological samples were collected for each days of gestation.

### Reverse transcription

Following the manufacturer's instructions, complementary DNA synthesis was performed on total RNA using SuperScript II Reverse Transcriptase (Invitrogen, Carlsbad CA, no. 18064-014). A reverse transcriptase negative control was used to ensure there was no DNA contamination.

### Real-time qRT-PCR

Transcript levels of *Kcnq1 *and *β-actin *were analyzed on the ABI Prism 7000 system (ABI, Foster City, CA). Reactions were conducted using the SYBR Green PCR Master Mix (ABI, Foster City, CA no. 4309155). On a 96-well plate, a reduced reaction of 20 μl was used instead of the suggested 50 μl indicated in the manufacturer's protocol. This included 10 μl of the SYBR Green reaction mix, a final concentration of 25 μM of each primer, the cDNA template and water to bring the final volume up to 20 μl. The PCR was performed under the following conditions: an initial denaturing step for 10 min at 95°C, an amplification step for 45 cycles of 95°C for 20 s, 55°C for 30 s and 72°C for 30 s, the final elongation step was at 72°C for 2 min. The *Kcnq1 *transcript was detected using the following left and right primers: 5'-CAAAGACCGTGGCAGTAAC-3' and 5'-CCTTCATTGCTGGCTACAAC-3'. The *Kcnq1 *transcript was normalized to *β-actin *using the following left and right primers: 5'-TGTTACCAACTGGGACGACA-3' and 5'-CCATCACAATGCCTGTGGTA-3'. Each qRT-PCR reaction was performed in triplicate with three biological replicates along with a no template negative control. The CT value of the *Kcnq1 *transcript was normalized to the CT value of the *β-actin *transcript. The standard deviation for each ratio was determined and the error bars represent 1 SD away from the average ratio.

### Allele-specific RT-PCR and quantification

The *Kcnq1 *transcript was amplified using Ruby Taq Master Mix (Affymetrix, Santa Clara, CA no. 71191) in a reduced 15 μl reaction using the following primers: 5'-CATCGGTGCCCGTCTGAACAGG-3' and 5'-TTGCTGGGTAGGAAGAGCTCAG-3'. PCR reactions were performed with experimental and control templates in parallel. Following the PCR, a restriction digest was performed with the *Nla*III (New England Biolabs, Ipswich, MA no. R0125) for 1 h at 37°C. PCR and digestion products were run on 7% polyacrylamide gels and quantified using the Kodak Gel Logic 2000 imaging system (Kodak, Rochester, NY). The relative paternal to maternal band intensities were calculated.

### 3C assays

3C was performed as described previously with the following modifications: tissues were homogenized and crosslinked with 1% formaldehyde. Before restriction enzyme digestion, all samples were subjected to RNAse treatment with a combination of RNAse A and RNAse H at 37°C for 20 minutes. Restriction digests were carried out with *Afl*III/*Nco*I. Digestion efficiency at each restriction site was determined by comparing amplification with primers spanning the restriction site to amplification with primers immediately downstream on the digested template. No restriction bias was observed across the region assayed by 3C (data not shown). PCR efficiency of each combination of primers was assessed with a control template prepared from two bacterial artificial chromosomes (BACs) encompassing the *Kcnq1ot1 *region analyzed, as described previously. The linear range for each primer pair was determined by serial dilution. PCR reactions were performed with experimental and control templates in parallel and PCR products were run on 7% polyacrylamide gels and quantified using the Kodak Gel Logic 2000 imaging system (Kodak, Rochester, NY). Crosslinking frequencies were calculated from duplicate PCR analyses of three independent 3C preparations. Interaction frequencies for *Kcnq1 *and *Kcnq1ot1 *were normalized to a control interaction at the *H19 *locus to allow comparisons between different tissues and cell types. A full list of primers is available upon request from the authors.

### Luciferase reporter assays

Enhancer candidates were amplified from mouse genomic DNA and subcloned into the pGL3-promoter vector (Promega, Madison, WI) upstream of the luciferase reporter. All clones were confirmed by sequencing. Constructs were screened by transfection of 100 ng pGL3 promoter vector and 10 ng pHRL basic vector/well (24-well plate) into NIH 3T3 cells with 1 μl Lipofectamine 2000/well (Invitrogen, Carlsbad, CA). Cell lysate was harvested the following day and *firefly *and *Renilla *luciferase activities were measured in 10 μl of each lysate using a Dual-Luciferase Reporter Assay System kit (Promega, Madison, WI) and *firefly *activity was normalized to *Renilla*. All transfections were performed in triplicate.

### Transgenic embryo assays

Enhancer candidate constructs were amplified from mouse genomic DNA, subcloned into the Hsp68-LacZ vector and transgenic mouse embryos were generated and stained (Cyagen Biosciences, Sunvale, CA). Images were obtained with an Olympus MVX10 stereoscope (Olympus, Center Valley, PA), cropped and levels adjusted with Adobe Photoshop (Adobe, San Jose, CA).

### Methyl-DNA immunoprecipitation

DNA from pooled mouse 7.5 dpc embryos and from mouse sperm was sonicated to obtain fragments ranging from 200 to 1,000 bp. An aliquot of DNA was saved for an input positive control. Me-DIP was performed using the Methylamp Methylated DNA Capture Kit (Epigentek, Farmingdale, NY)) and following the manufacturer's protocol. The *Kcnq1 *CpG Island was amplified using the following: 5'-CTGAGGGCAGCACGGTCTAT-3' and 5'-CTCCTGAGTCTCTCTTGTCACAACT-3'. *H19 *and *Kcnq1ot1 *promoters were used to control the quality of the immunoprecipitated substrates.

## Competing interests

The authors declare that they have no competing interests.

## Authors' contributions

NE was responsible for the development of the experiments, the 3C assays and associated analysis, and wrote and edited the manuscript. LK performed the allele-specific RNA analysis, qRT-PCR analysis, MeDIP experiments and helped edit the manuscript. AR generated the 3C substrates and helped perform the 3C assays. GB performed and analyzed the dual luciferase assays. All authors read and approved the final manuscript.
